# Ecofriendly Preparation of Rosmarinic Acid-poly(vinyl alcohol) Biofilms Using NADES/DES, Ultrasounds and Optimization via a Mixture-Process Design Strategy

**DOI:** 10.3390/ma17020377

**Published:** 2024-01-12

**Authors:** Beatrice Campanella, Mattia Simoncini, Elisa Passaglia, Francesca Cicogna, Gianluca Ciancaleoni, José González-Rivera, Luca Bernazzani, Emilia Bramanti

**Affiliations:** 1 National Research Council, Institute for the Chemistry of Organometallic Compounds, Via Giuseppe Moruzzi 1, 56124 Pisa, Italy; beatrice.campanella@pi.iccom.cnr.it (B.C.); mattia.simoncini@gmail.com (M.S.); elisa.passaglia@pi.iccom.cnr.it (E.P.); francesca.cicogna@pi.iccom.cnr.it (F.C.); 2 Department of Chemistry and Industrial Chemistry, University of Pisa, Via Giuseppe Moruzzi 13, 56124 Pisa, Italy; gianluca.ciancaleoni@unipi.it (G.C.); jose.gonzalezrivera@ino.cnr.it (J.G.-R.); luca.bernazzani@unipi.it (L.B.); 3 National Research Council, National Institute of Optics, Via Giuseppe Moruzzi 1, 56124 Pisa, Italy

**Keywords:** NADES, rosmarinic acid, bioactive materials

## Abstract

Green chemistry emphasizes the isolation of biologically active compounds from plants and biomass to produce renewable, bio-based products and materials through sustainability and circularity-driven innovation processes. In this work, we have investigated the extraction of rosmarinic acid (RA), a phenolic acid with several biological properties, from aromatic herbs using ultrasounds and low environmental risk natural deep eutectic solvents (NADES). Various solvent mixtures have been investigated, and the parameters influencing the process have been studied by a mixture-process experimental design to identify the optimal RA extraction conditions. The extraction yield has been calculated by HPLC-diode array analysis. The lactic acid:ethylene glycol mixture using an ultrasound-assisted process has been found to be the most versatile solvent system, giving RA yields 127–160% higher than hydroalcoholic extraction (70% ethanol). The deep eutectic solvent nature of lactic acid:ethylene glycol has been demonstrated for the first time by multi-technique characterization (^1^H-NMR and ^13^C-NMR, DSC, and W absorption properties). The aqueous raw extract has been directly incorporated into poly(vinyl alcohol) to obtain films with potential antibacterial properties for applications in the field of food and pharmaceutical packaging.

## 1. Introduction

The well-recognized biological activity of polyphenols makes them appealing molecules for the food, cosmetic, pharmaceutical, and nutraceutical industries [1]. Traditionally, polyphenols are extracted from plants by maceration with aqueous ethanol [2,3], but more recently, ultrasound-assisted extraction (UAE) and microwave-assisted extraction (MAE) are being increasingly proposed as alternative methods [4,5], alongside the replacement of petrochemical-derived solvents with more eco-friendly compounds [6]. Considering that polyphenols act as natural radical scavengers [2], which makes them prone to degradation and oxidation with subsequent alteration of their biological activity, the use of ultrasounds in UAE should therefore be carefully modulated in terms of time, frequency, temperature, power, and the solid/liquid ratio [7]. On the other hand, the use of microwaves for extraction is motivated mostly by the better ability to internally heat the medium in the case of polar solvents compared to the classic heat transfer by conduction [8]. This allows for a more rapid and homogeneous heat distribution in the medium.

Deep Eutectic Solvents (DESs) and Natural Deep Eutectic Solvents (NADESs) have been hypothesized to be alternative solvents for polyphenol extraction due to their cost-effectiveness, sustainability, and greater safety than traditional organic solvents [9]. DES/NADES are characterized by the presence of a hydrogen bond donor (HBD) and a hydrogen bond acceptor (HBA), which are not always uniquely identifiable. The mixing of HBD and HBA in suitable ratios generates liquid eutectic mixtures with high solubilization capability at room temperature. DES/NADES high viscosity could limit their potential applications; however, recently, they have been added to H_2_O, which not only lowers viscosities but also modulates the melting point and conductivity [10,11]. In some recent studies, the addition of water to DES/NADES for the extraction of phenolic compounds from natural sources, even in the case of UAE, has improved the final yields [12,13,14].

The main critical parameter associated with the use of DES/NADES in polyphenol extraction is the efficient back-recovery of products from the solvent, which is primarily carried out by solid–liquid extraction [15]. To avoid this additional step, the direct employment of the extract for the functionalization of the polymeric material could be a great improvement [16,17]. Thus, the employment of DES for the extraction of polyphenols contributes to their stabilization and could play a role as plasticizers and modify polymers’ mechanical properties [18,19].

**Figure 1:**
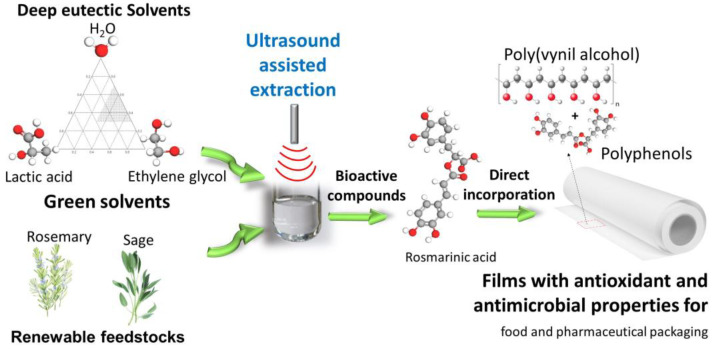
Overview of the full approach for the extraction of bioactive compounds by DES-UAE and its direct incorporation into polymeric films for smart biopacking applications.

The aim of this study has been to optimize the extraction of Rosmarinic acid (RA) from sage and rosemary using innovative low-impact natural compounds as solvents in tandem with UAE as an alternative extraction technique to maceration. RA is a secondary metabolite of many aromatic plants, including *Rosmarinus officinalis* L. and *Salvia officinalis* L. [20]. RA is the ester of caffeic acid and 3,4-dihydroxyphenylactic acid, it belongs to the category of polyphenols, and it has recognized antioxidant, antibacterial, antiviral, and anticarcinogenic properties. First, different solvent mixtures of natural compounds have been tested to identify the best-performing system in terms of the total amount of RA recovered. Then, full characterization of the optimized solvent system and their mixtures with water have been assessed by different techniques, i.e., ^1^H-NMR and ^13^C-NMR, DSC, and MW absorption properties. In the second part of the work, a mixture-process design was applied to define the optimal composition of the extraction mixture and optimize the experimental parameters of both extraction processes, i.e., traditional maceration and UAE, with the aim of maximizing the RA extraction yield from rosemary. The best extraction conditions of the proposed NADES-UAE process have thus been assessed in terms of the solid-to-liquid ratio, extraction time, and solvent composition. Finally, the extract has been successively used for the preparation of poly(vinyl alcohol) (PVA) films, which exploit the antioxidant and antimicrobial properties of RA itself and the other polyphenols present in the extract. The fabricated RA-PVA films may have promising applications in the field of food and pharmaceutical packaging. The full methodology and process strategy are illustrated in Figure 1.

PVA is a commercial, safe, non-toxic, inexpensive, biodegradable polymer approved by the FDA that possesses great mechanical properties such as semi-crystallinity and high-temperature stability, which could be exploited for further improvement of the work by preparing functional films using electrospinning [21]. PVA is used for protein purification, enzyme immobilization, membrane separation, pharmaceutical and cosmetics industrial use, and in several medical applications with satisfactory performances.

## 2. Materials and Methods

### 2.1. Materials

Rosmarinic acid (RA, 96%), methanol (MeOH for HPLC, ≥99.9%), sulphuric acid (ACS reagent, 95.0–98.0%), ethanol (EtOH for HPLC, ≥99.8%), lactic acid (LA, natural, ≥85%), ethylene glycol (EG, reagent grade, ≥99%), thymol (Tym), menthol (Me), glycine (Gly), betaine (Be), imidazole (Im), Folin-Ciocalteu’s phenol reagent, and gallic acid (GA, 97.5–102.5%) were purchased from Merck-Sigma Aldrich (Milan, Italy). All other chemicals used are of analytical grade unless otherwise stated. Deionized water was used throughout the experiments. Rosemary (*Rosmarinus officinalis* L.) and sage (*Salvia officinalis* L.) leaves were harvested from plants located in Valdicastello Carducci, Lucca (Italy), and immediately transferred to refrigerators to be stored at −80 °C. Before extraction, the leaves were washed with deionized water, lyophilized, and ground with a domestic blender.

### 2.2. Solvent Preparation and Characterization

The extraction mixtures were prepared by the thermal method by heating each binary mixture in a sand bath at 60–70 °C until a homogeneous liquid was formed (typically 0.5 to 3 h). For mixtures formed by two liquids, the components were mixed and stirred for 2 h at room temperature.

NMR spectra were measured using the pure liquids at 298 K on a JEOL JNM-ECZ500R 500 MHz spectrometer (JEOL Italia S.p.A., Milan, Italy). All the spectra were calibrated using an external reference (pure D2O). 1H NMR spectra were acquired with only 1 scan to ensure the quantity (gain = 1), while 13C NMR spectra were acquired with 32 scans to ensure a good signal-to-noise ratio. Deuterated benzene (C6D6) was used for instrument locking.

Infrared spectra were recorded by a Perkin-Elmer Spectrum One FTIR Spectrophotometer, equipped with a universal attenuated total reflectance (ATR) accessory and a triglycine sulfate TGS detector. For each sample, 32 scans were recorded, averaged, and Fourier-transformed to produce a spectrum with a nominal resolution of 4 cm^−1^.

Thermal stability was evaluated by thermogravimetric analysis (TGA) using a Seiko EXSTAR 7200 TGA/DTA instrument (SII NanoTechnology Inc., Tokyo, Japan). Measurements were carried out on 5–10 mg samples under air flow (200 mL/min) in the 30–600 °C range, with a 10 °C/min scanning rate.

Density was evaluated by weighing an exact volume of solvent (between 3 and 5 mL) with a microanalytical balance.

The films were characterized by spectrofluorimetric analysis using a Horiba FluoroMax-4 spectrofluorometer (Horiba Jobin Yvon Srl, Milan, Italy) (emission spectra: λ_exc_ = 330 nm, front entrance slit = 2 nm, front exit slit = 2 nm; emission spectra: λ_em_ = 430 nm, front entrance slit = 2 nm, front exit slit: 2 nm) and DSC measurements performed under a nitrogen atmosphere (50 mL/min) on a DSC-4000 (Perkin-Elmer, Waltham, MA, USA). The instrument was calibrated with indium (m.p. 156.6 °C, H = 28.5 J/g) and zinc (m.p. 419.5 °C). PVA samples were heated from 30 to 130 °C at 10 °C/min (1^st^ heating), cooled to 30 °C at the same scan rate (1^st^ cooling), and heated again to 130 °C at 10 °C/min (2^nd^ heating). The glass transition temperature (T_g_) was measured from the inflection point in the 2^nd^ heating thermogram.

The surface tension measurements were carried out with a Sigma703D tensiometer equipped with a De Noüy ring.

A homemade system was used for dynamic viscosity measurements [22]. Briefly, the viscometer consists of a PEEK capillary (i.d. = 0.25 mm, L = 75 cm) kept at room temperature and connected to a piezoelectric differential pressure transducer (P305D-20-2369, diaphragm 3-36, Validyne Northridge, CA, USA) capable of recording pressure differences in the 0–35 psi range. Viscosity measurements were carried out after the calibration of the viscometer with glycerol solutions in the 2.5–50% range by injecting 100 μL of solution. All data were collected at 20 kHz using a computer equipped with a data acquisition system (AT-MIO-16XE-50, National Instruments, Austin, TX, USA). Data collection and processing were performed using LabVIEW software (version 6.0, National Instruments).

Microwave (MW) absorption was evaluated by the temperature profile of a weighted quantity of solvent (100 mg) under a constant radiating power (10 W, 20 W, and 30 W) applied for 60 s. The MW was generated by a commercial SAIREM MW generator, Mod. GMP 03 K/SM, capable of delivering up to 300 W of radiating power with a frequency of 2.45 GHz. Temperature was measured using a fiber optic thermometer (Luxtron, model I652, with STF-1 fiberoptic sensor, Cuneo, Italy) placed in the sample.

The solid–liquid theoretical curves were determined using Equation (1), which represents the solid–liquid equilibrium curve:

(1) $$ln\left(\chi_{i}\cdot \gamma_{i}\right)=\frac{{∆}_{m}h_{i}}{R}\;\cdot \;\left(\frac{1}{T_{m,i}}-\frac{1}{T}\right)+\frac{\Delta_{m}{Cp}_{i}}{R}\;\cdot \;\left(\frac{T_{m,i}}{T}-ln\frac{T_{m,i}}{T}-1\right)$$

where *χ_i_* is the mole fraction of component *I*, *γ_i_* is its activity coefficient in the liquid phase (taken as 1 for ideal behavior), ∆*_m_h_i_* and *T_m,i_* are its melting enthalpy and temperature, respectively, Δ*_m_Cp_i_* is its heat capacity change upon melting, *R* is the ideal gas constant, and *T* is the absolute temperature of the system. This equation can be simplified by considering the heat capacity change upon the melting of a substance negligible, therefore Equation (2) was used:

(2) $$ln\left(\chi_{i}\cdot \gamma_{i}\right)=\frac{{∆}_{m}h_{i}}{R}\;\cdot \;\left(\frac{1}{T_{m,i}}-\frac{1}{T}\right)$$

### 2.3. Extraction Procedure

Maceration for the initial screening of the solvents was carried out for 1 h in a closed vessel placed in a thermostatically controlled bath at 50 °C with 10 mL of solvent and different solid/liquid (S/L) ratios. UAE was carried out using an ultrasonic processor (VCX750, power 750 W, 20 kHz; Sonics and Materials Inc., Newtown, CT, USA) equipped with a titanium alloy (Ti-6AI-4V) probe (13-mm diameter). Extraction was performed according to the conditions described in Section 2.4. The pulse sequence interval was 30 s ON and 30 s OFF in the whole extraction process. After extraction, the samples were diluted ten times with 50% ethanol and filtered by a 0.45 μm-pore-size cellulose membrane filter before HPLC analysis.

### 2.4. Mixture-Process and D-Optimal Design

Different proportions of the LA:EG:water mixture were investigated to maximize the extraction of RA. Specifically, a composite design was implemented by combining a simplex centroid design and a two-factor central composite design [23].

**Figure 2:**
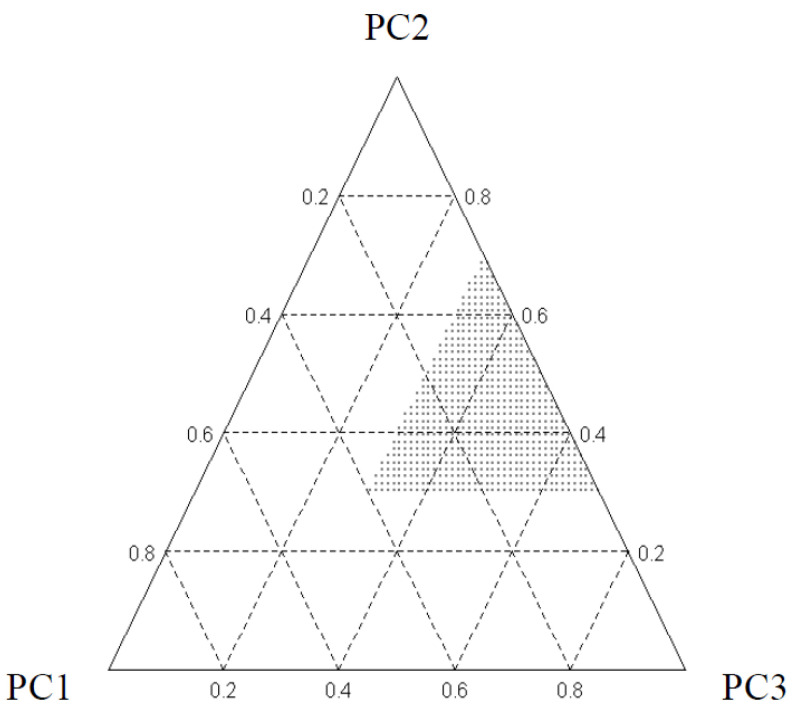
The pseudo components space and its limits. PC1 = water %, PC2 = LA %, PC3 = EG %.

The components of the extraction mixture were water (C1), LA (C2), and EG (C3), while the extraction time and the S/L ratio (for maceration) or the extraction time and the ultrasounds amplitude (for UAE) were selected as process variables. Considering preliminary experiences, some constraints were fixed to the extraction mixture composition: water ≤40%; LA and EG, ≥30% and ≤70%. Figure 2 shows the experimental region of interest, which appears as a triangular sub-region within the wider triangle according to the constraints imposed and constitutes the so-called space of the pseudo-components (PC1, PC2, and PC3). In addition, we set a constant extraction temperature equal to 50 °C for maceration and a maximum extraction temperature equal to 50 °C for UAE.

The combination of the two experimental designs (mixture design and central composite design) would give rise, for each extraction technique, to 63 experiments. A D-Optimal design was then applied to reduce the number of experiments to be performed, looking for the best compromise between the quality of the information to be obtained and the experimental effort. The experiments were chosen by an algorithm from all the possible combinations of the levels of the different factors to maximize the determinant of the information matrix, calculated as the product between the matrix of the experimental model and its transpose. The solution with 20 experiments was selected. Table S1 reports the details of the composite experimental plan. One of the twenty experiments (Exp #2) was replicated to estimate the experimental variance. Experimental design and statistical analysis were performed by means of the R-based software Chemometric Agile Tool (CAT) developed by the Italian group of Chemometrics and available online (http://gruppochemiometria.it/index.php/software, accessed on 8 January 2024) [24].

### 2.5. HPLC Analysis

RA in the extracts was quantified using an HPLC with a diode array detector. An HPLC gradient pump (P4000, Thermo Finnigan, Thermo Finnigan Italia S.p.A., Milan, Italy) was coupled with a vacuum membrane degasser (SCM1000, ThermoFinnigan), an AS3000 autosampler (Thermo Finnigan), a UV6000 diode array detector, and a FL3000 fluorescence detector (Thermo Finnigan). Separations were carried out using a reversed-phase HPLC column C18 Spherisorb S5 ODS2 (Waters, 250 mm × 4 mm, 5 µm). The column temperature was set at 40 °C and the injection volume was 2 μL. Mobile phases were 5% methanol–95% of 5 mM sulphuric acid in water (eluent A) and 95% methanol–5% of 5 mM sulphuric acid in water (eluent B). The gradient was as follows: 0–5 min, 80% A; 5–20 min, linear gradient up to 80% B; 20–22 min, linear gradient up to 100% B; 22–45 min 100% B. Post-run time was 15 min. Elution was performed at a solvent flow rate of 0.8 mL min^−1^. Detection was performed in absorbance at 220, 280, and 350 nm and in fluorescence setting the excitation and emission wavelengths at 280 and 365 nm, respectively. The ChromQuest™ Thermo Finnigan 4.2 Chromatography Data System was used to carry out HPLC-DAD/FD control, data acquisition, and data analysis.

### 2.6. PVA Films Preparation

The PVA solution was prepared by dissolving 0.5 g of PVA (146–186 kDa, 96% hydrolysis, Sigma Aldrich, St. Louis, MO, USA) in 10 mL of distilled water under magnetic stirring at 90 °C until complete dissolution. Increasing quantities (from 75 μL to 500 μL) of sage and rosemary UAE extract in 10% H_2_O–40% LA–50% EG were added to dissolved PVA. A mixture of 10% H_2_O–40% LA–50% EG was used for the preparation of a PVA reference film. The solutions were then poured into polyethylene sample containers and dried for 48 h at 37 °C to form the desired films. The films were finally removed by peeling and placing them in sealed containers to avoid moisture exchange.

## 3. Results and Discussion

### 3.1. Screening Step

**Figure 3:**
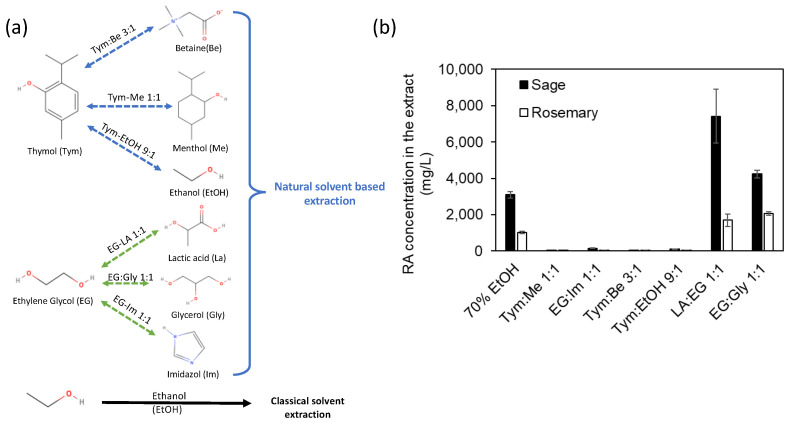
Composition of natural solvents (**a**) and plot of RA concentration found by HPLC analysis in rosemary and sage leaves extracts obtained by maceration using 70% EtOH, Tym:Me 1:1, Im:EG 1:1, Tym:Be 3:1, Tym:EtOH 9:1, LA:EG 1:1, and Gly:EG 1:1 (**b**). Error bars indicate the standard deviation of 3 replicates.

Choline chloride (ChCl) is generally considered the most widely used component for DES, although its use in vitro and in vivo experiments suggests that ChCl-based DESs may not be considered safe mixtures even if they consist of non-toxic compounds [25]. Instead, our approach for selecting the components of the extraction mixture was to use safe solvents with low environmental impact, possibly low cost, and obviously with the appropriate polarity and hydrophobicity characteristics. Based on these requirements, six solvent mixtures were screened for the extraction of RA from sage and rosemary leaves by maceration: Tym:Me 1:1, Im:EG 1:1, Tym:Be 3:1, Tym:EtOH 9:1, LA:EG 1:1, and EG:Gly 1:1 (Figure 3a).

For comparison purposes, 70% EtOH was also employed as a reference solvent. The results of the Folin assay show an average concentration of total polyphenols of around 150–160 mg GAE/g dry mass for both sage and rosemary extracts, regardless of the solvent used for the extraction. This may be ascribed to the poor specificity of the Folin test. For this reason, the following results are based only on HPLC-UV analyses.

The HPLC-UV profiles of the resulting extracts were qualitatively similar, and the differences in the intensity of RA peaks (Figure 3b) were compared with traditional alcoholic maceration by one-way ANOVA and Tukey’s post-hoc test (95% confidence level).

**Figure 4:**
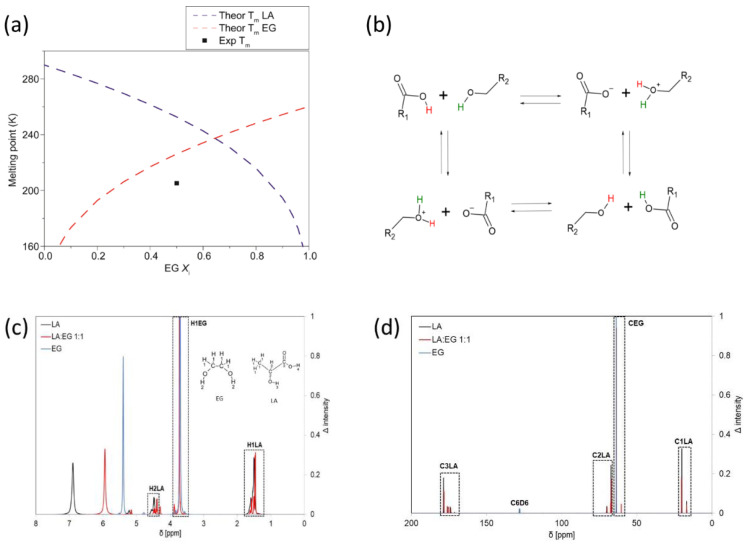
Experimental/theoretical solid–liquid phase diagrams for the LA:EG mixture (**a**), proposed exchange mechanism of H4AL and H2EG protons in the LA:EG mixture (**b**). [R1 = LA residue; R2 = EG residue], ^1^H-NMR spectra of LA (black), EG (blue), and LA:EG 1:1 (red) (**c**) and ^13^C-NMR spectra of LA (black), EG (blue), and LA:EG 1:1 (red) (**d**).

Extraction with LA:EG 1:1 and Gly:EG 1:1 gave yields statistically higher than extraction with 70% EtOH for both plants. The presence of LA seems to especially improve the content of RA. The acidity of LA could contribute to hydrolyzing the hemicellulose, cellulose, and pectin in the cell walls of the powdered plant leaves, thus facilitating the release and diffusion of RA from the plant matrix. Moreover, in previous studies, it was observed that an acidic environment is desirable for the stabilization of the extracted polyphenols [26,27]. The extractive efficiency of the LA:EG mixture was further proven by comparing the concentrations of RA obtained in this mixture (7400 mg/L and 1400 mg/L for sage and rosemary, respectively) with respect to those obtained in pure LA (3470 mg/L and 1000 mg/L for sage and rosemary, respectively) or pure EG (2320 mg/L and 1440 mg/L for sage and rosemary, respectively). These data seem to suggest a synergistic effect of the two solvents that contributes to the overall extractive efficiency of the system. Calorimetric analysis using DSC was performed to study the formation of a deep eutectic system between LA and EG. No phase transitions appear in the curve recorded between −80 and 50 °C for both heating and cooling (Figure S1), suggesting a melting point lower than −80 °C or kinetically hindered crystallization. To overcome the kinetic barrier, the liquid–solid transition was empirically estimated by cooling a small volume of the LA:EG 1:1 mixture in an acetone/dry ice bath. The phase transition was observed at −68 °C, which is significantly lower than the theoretical melting temperature of the mixture (−47 °C, see Experimental Details and Figure 4a), confirming that the LA:EG mixture can be defined as DES.

The LA:EG 1:1 mixture was also analyzed by ^1^H-NMR and ^13^C-NMR, and a hypothetical exchange mechanism is shown in Figure 4b. ^1^H-NMR spectra show that the chemical shifts of H1LA, H2LA, H3LA, and H1EG protons (H legend insert in Figure 4c) in pure compounds were substantially the same as those found in the 1:1 mixture. In the case of the acid proton of LA (H4LA) and the hydroxyl protons of EG (H2EG), a proton exchange takes place, involving the H4LA proton and two H2EG protons, as proven by the single signal observed for the mixture at around 6 ppm. The ^13^C-NMR spectra in Figure 4d show that C2LA, C3LA, and CEG carbon (C legend in the inset) are less shielded in their 1:1 mixture than in the pure components. This finding suggests the possibility that LA and EG are both H-bond donors. ^13^C-NMR spectra of the pure substances show a lower number of signals compared to their 1:1 mixture. Moreover, in the spectra of pure LA, a very intense peak is present around 178.6 ppm assigned to the carboxylic carbon of LA monomer, while at 175.6 ppm and 174.2 ppm, we can find two peaks with lower intensities assigned to the ester carbon of two different diastereomers owing to LA dimerization. At 173.8 ppm and 171.2 ppm, we also found two peaks that could indicate the ester carbon of two different LA trimers. From the literature, it is indeed known that even in dilute solutions, both the dimer and trimer of lactic acid are present [28]. In the ^13^C-NMR spectrum of the 1:1 LA:EG mixture, the signal ascribed to the carboxylic carbon of the LA monomer is de-shielded with respect to pure LA, for two signals due to the carbon ester of LA dimers and for two signals due to the ester carbon of LA trimers. At 176.1 ppm, however, the presence of a new peak is observed, which could be attributed to the formation of an ester bond between LA and EG. Other minor peaks could be due to the esters of the dimers and trimers of LA with EG.

In summary, NMR data suggest that the LA:EG mixture is a DES in which both components are H-bond donors, while the H-bond acceptors are the oxygen atoms of the esters between EG and LA, regardless of whether they were formed starting from the monomers, dimers, or trimers of the latter. Similar structures have been detected in inorganic ionic liquids based on chlorinated aluminum [29], while they have not yet been observed in organic DES with two simple components such as LA and EG. The esterification reaction between LA and poly-EG is reported in the literature for the synthesis of functional hydrogels [30].

**Figure 5:**
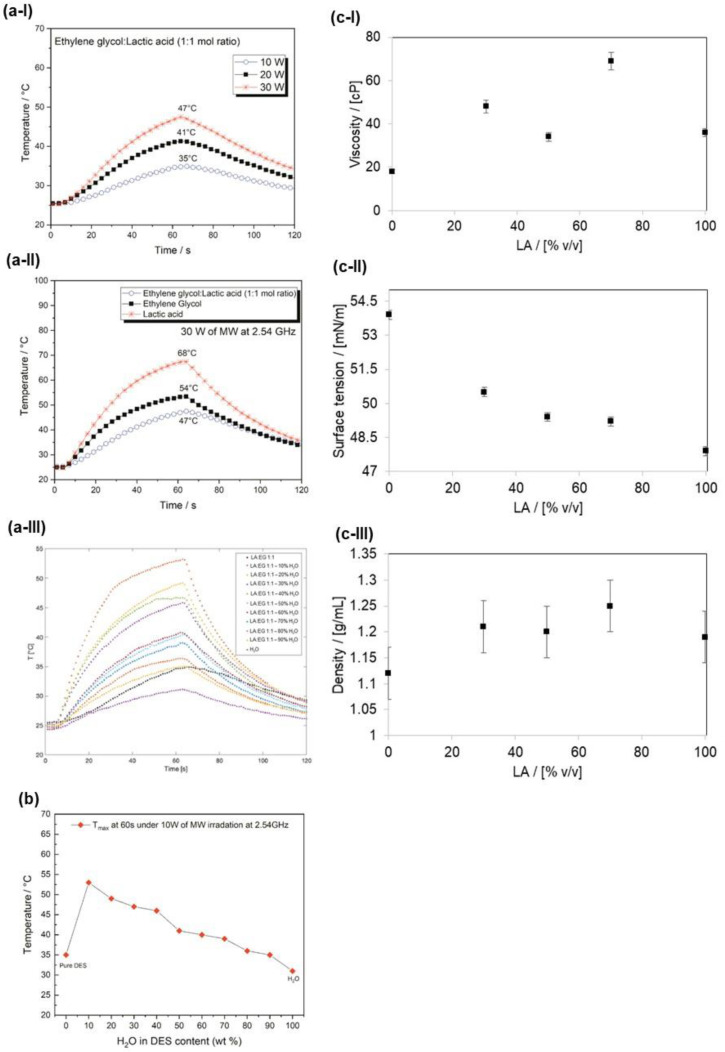
Trend of the temperature as a function of the irradiation time with different MW power (10, 20, and 30 W, from 0 to 60 s) and subsequent cooling (from 60 to 120 s) of LA:EG 1:1 (**a-I**), LA:EG 1:1, and their pure components using MW power = 30 W (**a-II**) and with various % of water (0–100%) and MW power = 10 W (**a-III**); trend of the T_max_ reached during the irradiation with MW as a function of the % of water in the mixture (**b**). Viscosity (**c-I**), Surface tension (**c-II**), and Density (**c-III**) of LA: EG mixtures.

The LA:EG solvent and the pure components (LA and EG) were further characterized through the absorption of MW, also with increasing concentrations of water. The MW absorption was evaluated by applying a constant radiating power (10 W, 20 W, and 30 W) for 60 s to a fixed amount of solvent (100 mg) and recording the temperature profile as a function of the irradiation time (Figure 5). The experimental setup guarantees a unidirectional MW field.

The absorption of MW depends on the presence of ionic species able to interact with the electromagnetic field generated by the MW source. When ChCl is used as HBA in polyalcohols-ChCl and organic acids-ChCl DES, we observed a strong MW absorption of DES due to its intrinsic capacity of forming hydrogen bond networks in the hydroxyl groups present and the ionic character of the HBA component (ChCl) [31]. The MW absorption of DES is higher than its pure components. A good MW response of LA:EG NADES was observed as a function of the MW irradiation power used (Figure 5(a-I)). However, the absorption of MW by LA:EG is lower than that of pure components, suggesting that the mixture has a poorer ionic character compared to pure components. This result fits the NMR findings well regarding the formation of esters between LA and EG (as discussed above), which heat up less efficiently with respect to the pure components (Figure 5(a-II)). The trend of the temperature observed by varying the content of water (Figure 5(a-III)) shows that for all ratios, the presence of water produces an increase in temperature, which is higher for a low content of water, in agreement with the trend of the DES above mentioned in which a water concentration greater than 40% *v*/*v* could deconstruct the complex structure typical of DES (see Figure 5b).

Overall, it is reasonable to hypothesize that the remarkably different MW heating responses of LA:EG, LA:EG:H_2_O, and ChCl-based DES can be associated with a different hydrogen bond network arrangement promoted by both the ionic character of the different HBAs and the presence of water. At a low content, water molecules can take part in the superstructure generated by HBA hydrogen bond interactions, improving the MW-induced heating response; at a higher content, hydrogen bonds between water and HBA overpower those between HBA, destroying the network and the eutectic fluid structure.

LA and EG mixtures in various proportions were also analyzed from a rheological point of view. Figure 5 shows the results of viscosity (5c-I), surface tension (5c-II), and density (5c-III) measurements. The surface tension of the mixture decreases as the content of LA increases, while the trend of viscosity and density are mutually consistent, but they are not monotonous as expected from a liquid–liquid mixture. Similar trends were instead found in the inorganic ionic liquids previously mentioned [29], which share with the LA:EG mixtures the presence of complex structures with dimers and trimers of the pure components, as observed in the NMR analysis.

### 3.2. Optimization: Mixture-Process Design

**Figure 6:**
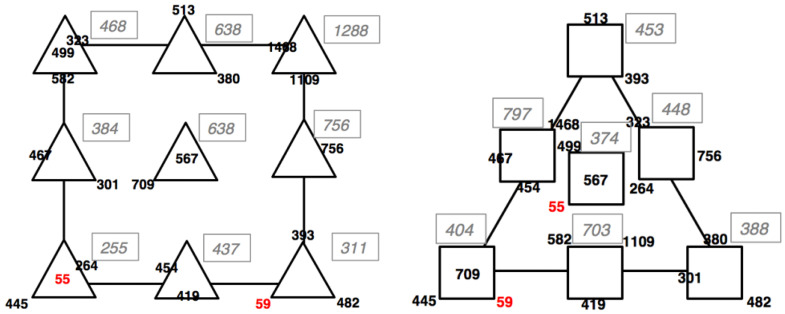
Experimental responses in the space of process variables (on the **left**) and of mixtures (on the **right**), respectively, for the maceration process optimization. Black numbers are the responses of each single experiment, grey italic numbers are the averages of the responses of the experiments, and red numbers are potential outliers.

A mixture-process design was applied to define the optimal composition of the LA:EG mixture and the experimental parameters of the maceration process (S/L ratio and time) to maximize the extraction of RA from rosemary. In each experiment, the concentration of RA was measured by HPLC-DAD analysis as a response. Figure 6 shows the results of 20 experiments in the space of process variables and mixtures, respectively.

The results suggest that the process variables mostly affect the system if compared with the composition of the extraction mixture, with the mean responses changing over a range in the domain of the process variables more than in the mixture space. The coefficients of the mathematical model were calculated by multiple linear regression. The mathematical model to be maximized is:

Y = 548 PC1 (*) + 476 PC2 (*) + 443 PC3 (*) + 980 PC1PC2 + 716 PC1PC3 + 119 PC2PC3 − 4715 PC1PC2PC3 + 200 X1 (*) + 204 X2 (**) + 141 X1X2 + 81 X12 − 93 X22(3)

where:

- PC1 = conc% *v*/*v* H2O.
- PC2 = conc% *v*/*v* LA.
- PC3 = conc% *v*/*v* EG.
- X1 = S/L ratio.
- X2 = time.

The values of the coefficients associated with the variables are shown in Equation (3), which also considers their variance and significance reported using asterisks (* = *p* < 0.05; ** = *p* < 0.01). The overall explained variance is 53%. Only five linear terms were significant, and all have positive values. This indicates that the response increases as the values of these factors increase.

This model showed a good fit of the experimental data.

To obtain the best extraction conditions from the response surfaces, the individual models must be calculated respectively in the space of the process and the mixture variables. The maximization of RA extraction yield is obtained for mixtures with a water content equal to 20%, which indicates the highest significance of the viscosity of the mixture for greater extraction efficiency. Considering the process and the mixture variables together, S/L ratio = 1/10, 45 min extraction time, 20% H_2_O/50% LA/30% EG = 30% mixture composition were the best conditions found.

**Figure 7:**
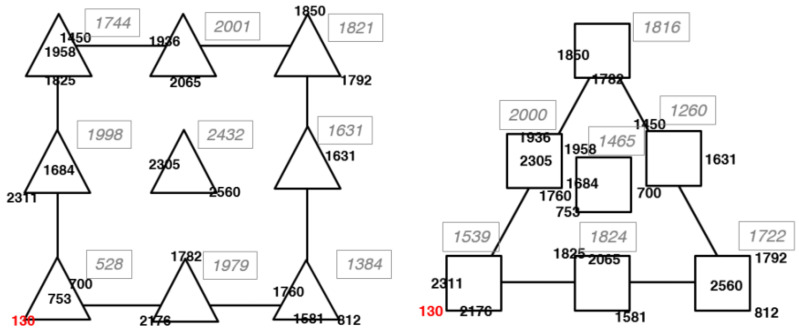
Experimental responses in the space of process variables (on the **left**) and of mixtures (on the **right**), respectively, for the US process optimization. Black numbers are the responses of each single experiment, grey italic numbers are the averages of the responses of the experiments, and red numbers are potential outliers.

The same experimental plan was applied to optimize RA extraction from rosemary by ultrasounds. Also in this case, we found that the process variables affect the system more than the LA:EG mixture composition (Figure 7).

The coefficients of the mathematical model, calculated by multiple linear regression, were the following:

Y = 2731 PC1(**) + 2700 PC2(**) + 22349 PC3(**) − 1181PC1PC2 − 192 PC1PC3 − 849 PC2PC3 + 6783 PC1PC2PC3 + 194 X1 + 330 X2(*) − 254 X1X2 − 563 X12 − 572 X22 (4)

Only five linear terms were statistically significant and all of them have positive values. This indicates that the response increases as the values of these factors increase. The highest extraction yields were obtained for LA:EG mixtures with the maximum content of water (%) and LA (%). In the space of process variables, RA concentration increases for the intermediate values of the two variables. For UAE, the optimization of the extraction process requires a compromise between two factors: The extraction efficiency of RA and its degradation as both increase with the extraction time and US amplitude. The degradation of RA could be indeed ascribed to the US cavitation bubbles as a potential radical source. Considering the process and mixture variables globally, 30% US amplitude, 30 min extraction time, and a 20% H_2_O/30% LA/50% EG = 50% mixture were the best operating conditions found.

Compared to a traditional extraction with the 50% LA/50% EG mixture (i.e., the starting condition of this work), the quantity of RA extracted was 1.78 and 4.6 times higher with the optimized maceration and ultrasound extraction protocols, respectively. In addition to providing a higher yield, ultrasound extraction reduces the extraction time from 45 to 30 min.

### 3.3. Inclusion of the Extract into PVA Films

**Figure 8:**
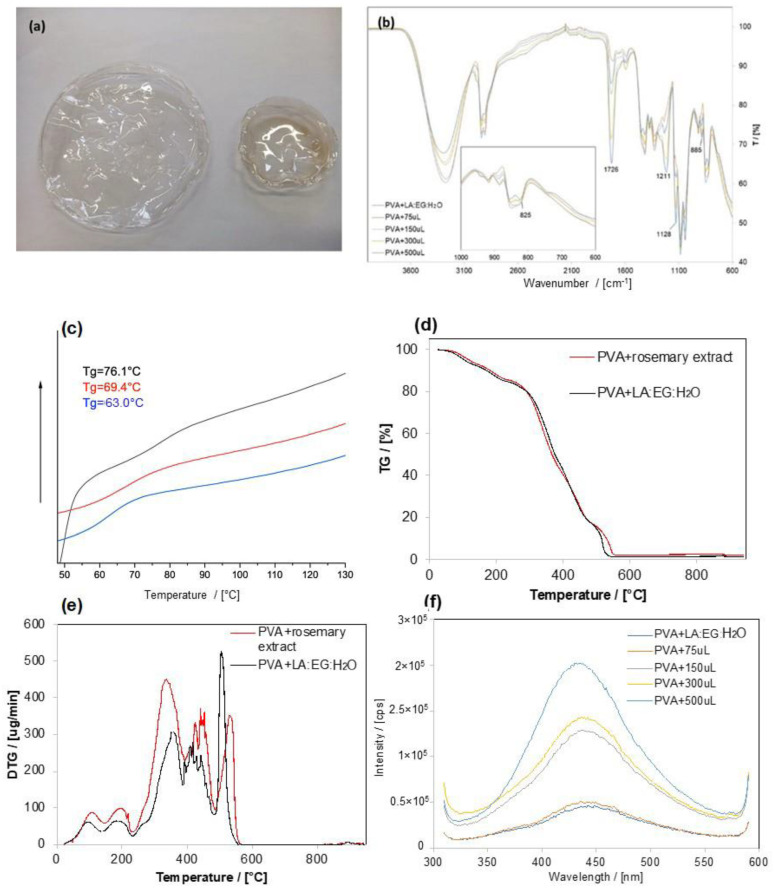
(**a**) Pictures of PVA film (left) and film containing 300 μL of rosemary extract in LA:EG:H_2_O (left); (**b**) FTIR-ATR spectra and magnification of the region between 1000 and 750 cm^−1^ of PVA samples with 150 μL of LA:EG:H_2_O incorporated and containing increasing quantities (from 75 μL to 500 μL) of rosemary extracts); (**c**) DSC curves of films of starting PVA (black curve) and sample containing LA:EG:H_2_O (blank, red curve) and 300 μL (blue curve) of rosemary extract; TGA (**d**) and DTG (**e**) in air of a PVA film containing 150 μL of LA:EG:H_2_O and a PVA film containing 150 μL of rosemary extract (RA in the film of PVA equal to 17.9 mg/g dry mass); (**f**) emission (λ_exc_ = 330 nm) spectra relative to the PVA films with 150 μL of LA:EG:H_2_O solution and increasing quantities of rosemary extract (75–500 μL) (front Entrance Slit: 2 nm; Front Exit Slit: 2 nm).

The incorporation of rosemary extracts in PVA produced transparent and glossy films with a light brown color by increasing the extract content (Figure 8a). The chemical characterization and stability of this material are preliminary to any employment as a functional material with potential antibacterial and antioxidant properties for food packaging. For these reasons, the films (with and without rosemary extract) were characterized by ATR-FTIR, UV-Vis, DSC, and thermogravimetric analyses (Figure 8). In the ATR-FTIR spectra of PVA films prepared with increasing quantities of rosemary extract, in addition to the characteristic bands of PVA, LA, and EG, the skeletal vibrations of the aromatic ring at 820 cm^−1^ are clearly visible [32], with intensity proportional to the amount of extract, confirming the increased content of RA (Figure 8b).

The quantitative determination of RA in the PVA films was assessed using UV-Vis spectroscopy. Figure S2 shows the absorbance at 330 nm as a function of the mass of extract expressed in grams. By comparing the calibration curve of the RA standard and the curve related to functionalized films, it was possible to determine the concentration of RA after measuring the thickness of the films, which was found to be 0.30 mm.

In order to evaluate RA stability in the PVA film, the same samples were also analyzed by UV-Vis spectroscopy after three-month aging at room temperature and under normal lighting conditions. We observed that the absorption at 330 nm after three months was not significantly different compared to fresh samples, except for samples with a higher quantity of extract, which showed a decrease of approximately 25% (Figure S2). DSC measurements were carried out to evaluate the effects of extract addition on the thermal characteristics of PVA films (Figure 8c). A decrease in T_g_ values was observed, as expected, due to the plasticizing effect of the LA:EG mixture containing rosemary extract.

Figure 8d shows the TG curve and its derivative (Figure 8e) in air of PVA films designed with 150 µL of the LA: EG mixture (reference sample) and 150 µL of the rosemary extract. The first peak in both PVA films around 100 °C can be ascribed to the evaporation of the water included in PVA. The LA:EG mixture has a degradation peak around 180 °C. As described by Shaulov et al. [33], the degradation of PVA occurs through several steps that include hydroxyl groups, which are dehydrated and form double bonds—primary polyenes (Stage I at 230–300 °C)—and, subsequently, the further dehydration in the remaining hydroxyl sites or the formation of macroradicals starting from the carbonaceous backbone, which is reduced to form only a carbonaceous residue (Stages II and III between 300–400 and 400–480 °C, respectively). As the analysis was conducted in air, the carbon residue was further oxidized to CO_2_ (Stage IV at 480–600 °C).

In the modified PVA film, Stage I occurs at lower temperatures (335 °C vs. 353 °C), Stages II and III are delayed by approximately 10 °C, and Stage IV is delayed from 504 to 531 °C. The antioxidant action of RA and other polyphenols present in the extract can be presumably detected around Stage IV.

Finally, the emission spectrum of the functionalized PVA film showed a characteristic peak of RA at 430 nm (Figure 8f) [34], which, once again, depends on the amount of rosemary extract added. Indeed, the initial findings presented in this study indicate that the photophysical and thermal properties of PVA films can be adjusted by adding rosemary extract directly into the eutectic mixture. Different quantities of rosemary extract can be used to produce films with good filming ability, adjustable flexibility (T_g_), high thermal stability, and fluorescence characteristics suitable for use as a tracer for quality control in food packaging.

## 4. Conclusions

The employment of the new LA:EG DES was proposed for the green US-assisted extraction of RA from rosemary and sage. The deep eutectic solvent nature of the LA:EG mixture has been demonstrated for the first time by multi-technique characterization (1H-NMR and 13C-NMR, DSC, and MW absorption properties). A mixture-process design combined with a D-optimal approach to reduce the number of experiments employed and optimize the proportions of the components. The LA:EG:water mixture at the molar ratio of 3:5:2 resulted in the highest efficiency for US extraction. The optimized blend enabled the extraction of 17.9 mg of RA/g dry mass from rosemary and 29.7 mg of RA/g dry mass of sage in 30 min with 160% and 127% yields, respectively, which are higher than traditional extraction with organic solvents. The homogeneous dispersion of polyphenols in PVA indeed induces the addition of antioxidant and antibacterial features, making the resulting composite films powerful for both food packaging and biomedical applications [35]. In this work, the extract was directly incorporated into pre-polymerized PVA, obtaining films stable against heat and aging. Further investigation will be addressed to evaluate the interaction between the components of the natural extract and the polymeric chains, the structural changes in the film, and its mechanical and biological properties.

## Data Availability

The data that support the findings of this study are available from the corresponding author upon reasonable request.

## Supplementary Materials

The following supporting information can be downloaded at: https://www.mdpi.com/article/10.3390/ma17020377/s1, Table S1. Experimental plan of the mixture-process design; Figure S1. DSC analysis of the LA:EG 1:1 mixture. Blue line: cooling down; green line: isotherm; red line: heating; Figure S2. Absorbance at 330 nm of fresh and 3-month-aged PVA films containing increasing quantities (from 75 to 500 μL) of rosemary extract.
