# Supplementary material for: Ecofriendly Preparation of Rosmarinic Acid-poly(vinyl alcohol) Biofilms Using NADES/DES, Ultrasounds and Optimization via a Mixture-Process Design Strategy
Source: Materials (Basel). 2024 Jan 12;17(2):377. doi: 10.3390/ma17020377 (PMC10820272; doi:10.3390/ma17020377)
Supplement: Supplementary file 1 [file materials-17-00377-s001.zip › materials-2799696-supplementary.pdf]

SUPPLEMENTARY MATERIAL

Ecofriendly preparation of rosmarinic acid-poly(vinyl alcohol) biofilms using NADES, ultrasounds and optimization *via* a mixture-process design strategy

Beatrice Campanella[1], Mattia Simoncini[2], Elisa Passaglia[1], Francesca Cicogna[1], Gianluca Ciancaleoni[2], Josè Gonzalez Rivera[3], Luca Bernazzani[2], Emilia Bramanti[1,*]

[1] National Research Council, Institute for the Chemistry of Organometallic Compounds, via Giuseppe Moruzzi 1, Pisa, 56124, Italy

[2] University of Pisa, Department of Chemistry and Industrial Chemistry, via Giuseppe Moruzzi 13, Pisa, 56124, Italy

[3] National Research Council, National Institute of Optics, via Giuseppe Moruzzi 1, Pisa, 56124, Italy

*Corresponding authors: emilia.bramanti@cnr.it

Table of contents da rivedere

Table S1	……………………………………………….	2
Figure S1	……………………………………………….	3
Figure S2	……………………………………………….	4

Table S1. Experimental plan of the mixture-process design.

exp	PC1	PC2	PC3	X1	X2
#1	1	0	0	-1	-1
#2	0.333	0.333	0.333	-1	-1
#3	0	0.5	0.5	-1	-1
#4	1	0	0	0	-1
#5	0	1	0	0	-1
#6	0	0	1	1	-1
#7	0.5	0	0.5	1	-1
#8	0.5	0.5	0	1	-1
#9	1	0	0	-1	0
#10	0.333	0.333	0.333	-1	0
#11	0.5	0.5	0	0	0
#12	0	0	1	0	0
#13	0	0.5	0.5	1	0
#14	0.5	0	0.5	-1	1
#15	0.333	0.333	0.333	-1	1
#16	0	0.5	0.5	-1	1
#17	0.5	0	0.5	0	1
#18	0.5	0.5	0	0	1
#19	0	1	0	1	1
#20	0	0	1	1	1

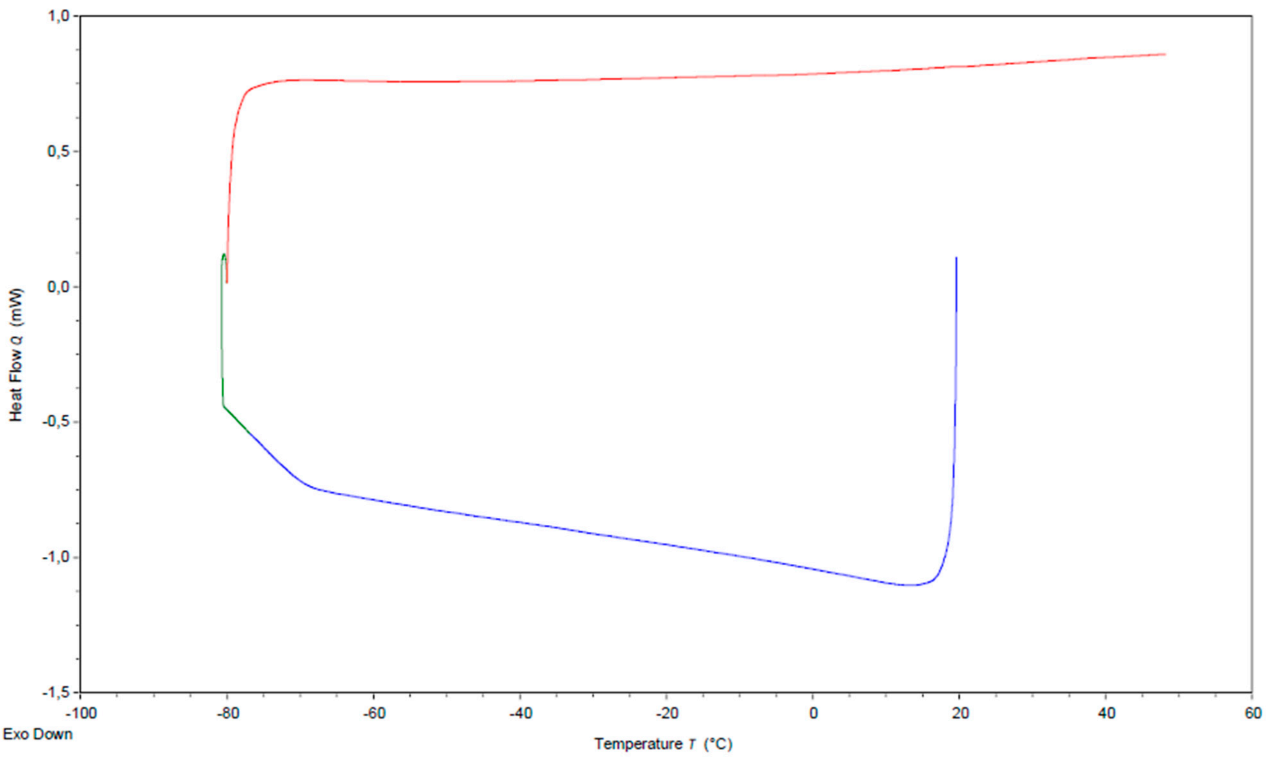

Figure S1. DSC analysis of the LA:EG 1:1 mixture. Blue line: cooling down; green line: isotherm; red line: heating.

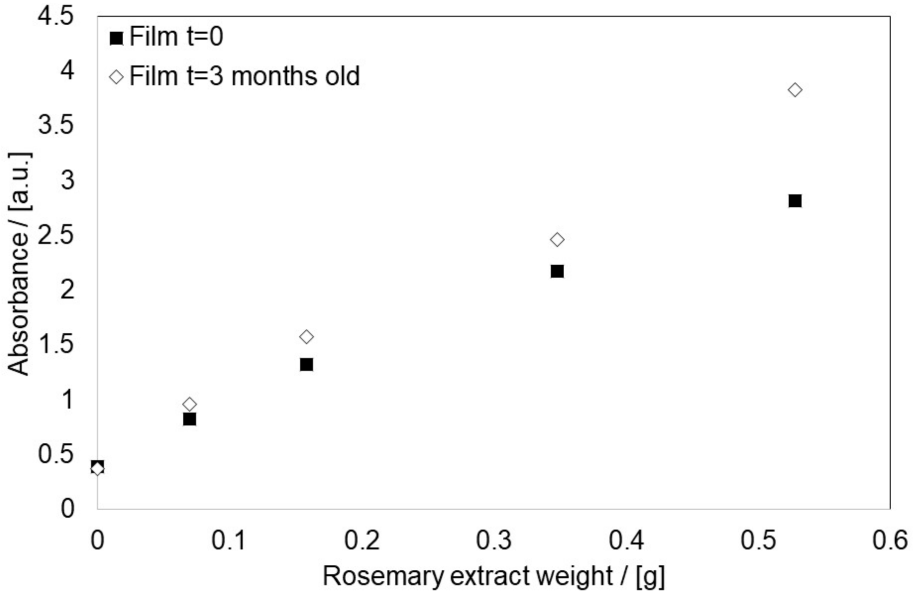

Figure S2. Calibration curve at 330 nm of fresh and 3 months old PVA films containing increasing quantities (from 75 μL to 500 μL) of rosemary extract.
